# Was There Land on the Early Earth?

**DOI:** 10.3390/life11111142

**Published:** 2021-10-26

**Authors:** Jun Korenaga

**Affiliations:** Department of Earth and Planetary Sciences, Yale University, New Haven, CT 06511, USA; jun.korenaga@yale.edu

**Keywords:** exposed land, continents, ocean islands, wet–dry cycles

## Abstract

The presence of exposed land on the early Earth is a prerequisite for a certain type of prebiotic chemical evolution in which the oscillating activity of water, driven by short-term, day–night, and seasonal cycles, facilitates the synthesis of proto-biopolymers. Exposed land is, however, not guaranteed to exist on the early Earth, which is likely to have been drastically different from the modern Earth. This mini-review attempts to provide an up-to-date account on the possibility of exposed land on the early Earth by integrating recent geological and geophysical findings. Owing to the competing effects of the growing ocean and continents in the Hadean, a substantial expanse of the Earth’s surface (∼20% or more) could have been covered by exposed continents in the mid-Hadean. In contrast, exposed land may have been limited to isolated ocean islands in the late Hadean and early Archean. The importance of exposed land during the origins of life remains an open question.

## 1. Introduction

Liquid water, the medium of biology, moonlights as the chemical nexus of biochemistry (e.g., [1,2]). Water is by far the most frequent and abundant metabolite in biology [3]. Between a third and a half of known biochemical reactions consume or produce water. All of today’s universal biopolymers and most metabolites are synthesized by condensation–dehydration reactions and are degraded and made available for recycling by hydrolysis [4]. A simple explanation for the centrality of water as both the medium and primary reactive species in biology is that prebiotic chemical evolution was governed by the chemistry of water. It has been proposed that cycling water activity related to short-term (minutes to hours), day–night, and seasonal cycles drove both the synthesis and degradation of proto-biopolymers during prebiotic chemical evolution [5,6,7,8]; the oscillating activity of water on land surfaces of our rotating planet causes oscillations in the directionalities of water-based chemical reactions and may have driven prebiotic chemical evolution. Such cycling of water activity requires exposed land mass. Simple thermodynamic considerations based on Le Chatelier’s principle indicate that the absence of exposed land would cause the total hydrolysis of all polymers and metabolites (e.g., [9,10]).

Was there exposed land during the origins of life, or was the early Earth completely covered by an ocean? This question is important to our understanding of the origins of life [11,12,13,14] but is very difficult to answer either observationally or theoretically. The availability of geological records diminishes as we go deeper in time, because plate tectonics recycles surface materials to the interior by subduction [15,16]. Although evidence of exposure has been identified back to Paleoarchean sequences such as the 3.48 Ga Dresser Formation [17], the 3.33 Ga Josefsdal Chert [18], and the 3.26 Ga Mendon Formation [19,20], there is no accepted or definitive record for regionally expansive exposure horizons prior to 3.0 Ga [21].

However, the lack of a geological record does not mean that there was no exposed land. Only ∼8% of the present-day continental crust is of Archean ages (i.e., from 2.5 to 4.0 Ga) [22]; if the mass of the continental crust reached the present level by ∼4 Ga, as recent crustal growth models suggest [23,24], more than 90% of the Archean crust would not have been preserved. Even if expansive exposed land existed before 3 Ga, it could have been easily destroyed by plate-tectonic recycling. Virtually no crust is of the Hadean age (before 4.0 Ga) [25,26,27], and zircons of the Hadean age are extremely rare, with most of them from a single locality (Jack Hills in Western Australia) [28,29]. Inferring the spatial extent of exposed land from Hadean zircons (e.g., [30]) is thus extremely challenging, unless significantly more localities of Hadean zircons can be documented [29]. It is important to understand that the spatial extent of exposed land differs from that of continents, as the former refers to only the part of continents above the sea level (with contributions from oceanic islands); this distinction is not always appreciated in the literature (e.g., [31]).

The amount of exposed land can be estimated from theoretical considerations, but we need to understand that such theoretical approaches involve a number of important assumptions (e.g., [21,32,33,34,35,36,37]). At the most fundamental level, the extent of exposed land is determined by the volume of surface water and the amplitude of topographic variations; we do not expect much exposed land in the case of a deep ocean or minute topographic variations. The volume of surface water can change with time, owing to degassing and regassing caused by mantle convection (e.g., [38,39,40,41,42]). The deep water cycle at the present suggests that regassing is more efficient than degassing, resulting in a gradual reduction in ocean volume [38,43]. Thus, the volume of the ocean is likely to have been greater in the past [41]. At the same time, however, the dynamics of magma ocean [44,45] suggests that most water must have been trapped in a solidifying magma ocean [46,47], implying a shallow ocean degassed from the solidifying mantle in the very early Earth. Thus, the history of ocean volume is probably non-monotonic.

**Figure 1:**
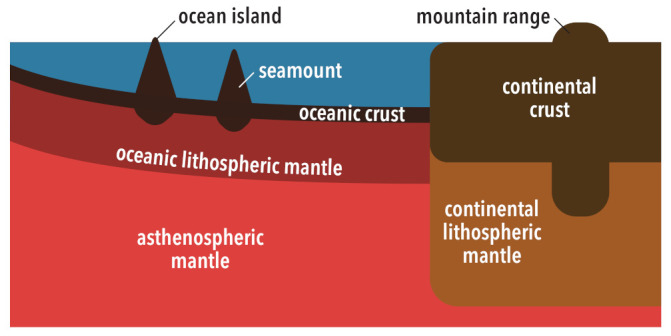
Schematic illustration for the present-day chemical structure of oceanic and continental domains. Continental crust is much thicker and slightly less dense than oceanic crust. Both kinds of crust are underlain by lithospheric mantle, which is less dense than asthenospheric mantle. Continental lithospheric mantle and oceanic lithsopheric mantle are similar in chemistry, but the former is generally less dense than the latter. “Oceanic lithospheric mantle” here refers to the residual mantle after the melting beneath mid-ocean ridges, and it must be distinguished from a similar term “oceanic lithosphere”, which is usually a synonym for a top thermal boundary layer of mantle convection and grows with time by conductive heat loss from the surface. The illustration is not drawn to scale, except for the lateral extent of these two domains. At present, oceanic crust is about 7 km thick and continental crust is about 40 km thick on average. Oceanic lithospheric mantle is about 70 km thick, and continental lithospheric mantle is about 200 km thick. Seafloor is about 2.5 km deep when it is created at mid-ocean ridges and becomes 5–6 km deep after 100 million years. The contact between the oceanic and continental domains shown here corresponds to those expected at passive margins, such as those in the Atlantic; oceanic crust and lithospheric mantle subduct at active margins, such as those around the Pacific.

When considering topographic variations, it is convenient to divide Earth’s surface into the oceanic and continental domains (Figure 1), as they are characterized by very different subsurface structures (e.g., [48,49,50,51]). Generally, the continental domain is more buoyant than the oceanic domain, so it could potentially provide a vast amount of exposed land as on the present-day Earth. However, the structures of these two domains change with time, primarily because Earth is cooling [52,53]; their relative buoyancy does not stay constant. The extent of Earth’s surface occupied by the continental domain also changes with time (e.g., [23,54,55,56,57,58,59,60]). In addition to this global-scale ocean–continent dichotomy, each domain can exhibit topographic variations, e.g., hotspot islands and oceanic plateaus for the oceanic domain and mountain ranges for the continental domain. The abundance of hotspot islands and oceanic plateaus can be related to the intensity of mantle plumes (e.g., [61,62]), which in turn is affected by the cooling history of the core [63,64]. The height of mountain ranges is regulated by the strength of the continental crust [65,66], which was weaker in the past due to the greater amount of radiogenic heating. Large-scale topographic variations across ocean basins, known as seafloor subsidence, determine the water-holding capacity of the ocean basins [67], and the rate of seafloor subsidence is controlled by the tempo of plate tectonics [68,69].

Thus, to estimate the amount of exposed land on the early Earth, we need to understand how the entire Earth system, including the core, mantle, crust, ocean, and atmosphere, may have behaved, starting from the magma ocean stage. Naturally, such an estimate is highly speculative. There is still no consensus, for example, even about when plate tectonics initiated on Earth (e.g., [23,70,71,72,73,74,75,76,77,78]). However, no component in Earth’s system exists in isolation. Additionally, the beginning of the early Earth is constrained by the formation history of Earth, and the early Earth had to evolve into the familiar modern Earth. Such interdependence and temporal continuity can be exploited to identify a likely scenario for the evolution of the early Earth landscape that makes sense in terms of basic physics and chemistry and is consistent with available observational constraints [79]. Given the fragmentary nature of geological data pertinent to the early Earth, such a synthesis with a sound geodynamical foundation is of critical importance. Previous reviews on the nature of the early Earth, in particular, the Hadean Earth, tend to be compromised by the lack of a thorough understanding of the physics of magma oceans and mantle convection (e.g., [76,80,81,82]); this is not surprising because there have been quite a few conflicting geodynamical studies (e.g., [83,84,85,86,87,88,89,90,91,92]), the clarification of which has become available only recently [46,79,93,94,95,96,97]. In what follows, I first describe what I consider as the most likely scenario in a narrative form, which is intended to be digestible for non-experts of earth sciences. To maximize readability, caveats are minimized. Then, I provide somewhat detailed accounts on how each segment of the scenario may be defended, including a critique on a recent review article by Russell [14], who argues that the Hadean Earth was a water world; this part is more technical and is meant for those who are reasonably familiar with earth sciences. This technical part is still kept brief for readability; interested readers are encouraged to consult the cited studies for fuller expositions.

## 2. A Most Likely Scenario

The theory of planetary formation suggests that the final stage of Earth formation involves multiple giant impacts, the last of which is the so-called Moon-forming giant impact [98]. It is commonly thought that, in the Moon-forming giant impact, a Mars-sized impactor hit the proto-Earth, and the deposition of an enormous amount of kinetic energy resulted in the formation of a deep magma ocean [99]. The details of this last giant impact are still much debated [100,101,102,103], but the formation of a global magma ocean is difficult to avoid. Due to the low viscosity of magma, a magma ocean convects vigorously [104]. Such a vigorous convection helps a magma ocean (and also the core in case of whole-mantle magma ocean) cool down quickly, and the life-time of a magma ocean is quite short, terminating potentially after only a few tens of thousands of years [97]. Some details of how a magma ocean solidifies are important. As the liquidus of the silicate mantle increases with pressure more rapidly than the adiabat of the mantle [105], a magma ocean starts to solidify from the bottom up [45,97]. Additionally, after a magma ocean starts to solidify, its solid fraction increases from 0% to ∼60% very quickly, after which the mixture of solid and melt becomes rheologically solid [44]. That is, at a solid fraction greater than ∼60%, solid grains are in contact, and the viscosity of the mixture is governed by the viscosity of solids. Viscosity increases by more than twenty orders of magnitude at this rheological transition. As a magma ocean starts to solidify from the bottom up, and because a solidifying magma becomes solid after ∼60% solidification, the cooling of the core slows down considerably during the early stage of magma ocean solidification [97].

**Figure 2:**
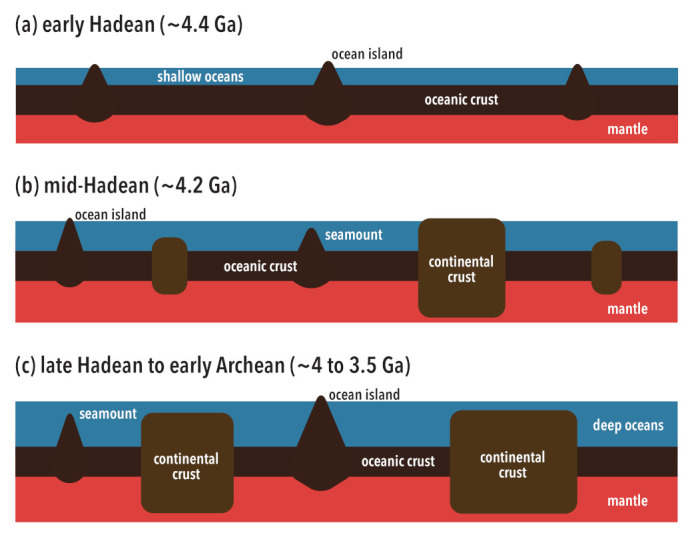
A possible evolution of early Earth landscape. (**a**) In the early Hadean, the ocean was likely shallow (∼1 km) because most of water was yet to be degassed from the mantle. Continental crust was absent, but the activity of mantle plumes was probably high to create quite a few ocean islands. (**b**) In the mid-Hadean, a substantial mass of continental crust started to appear. The growth of continents could outpace that of ocean, and some fraction of continents could have been exposed. Ocean islands continued to provide exposed land though they may not have existed in abundance. (**c**) Around the late Hadean to the early Archean, the mantle is likely to have been full degassed, resulting in a deep ocean. Even with the mass of continents grown to the present-day level, continents were below the sea level, making ocean islands the sole source of exposed land. In this illustration, distinction between lithospheric and asthenospheric mantle is omitted for simplicity. Additionally, the operation of plate tectonics is assumed at all panels, but the subduction of oceanic crust is omitted for simplicity.

The rheological transition in a solidifying magma ocean has important consequences for the early atmosphere. Water is much more soluble in magma than carbon dioxide [106,107,108], and at a solid fraction of ∼60%, most of the water is contained in the melt phase of a solidifying magma ocean, whereas most of carbon dioxide is in the atmosphere. Water contained in the melt phase remains mostly trapped within the mantle during the subsequent solidification of magma oceans [46,47]. Thus, the early ocean was likely to have been shallow (<1 km) and was covered by a Venus-like, massive CO${}_{2}$-rich atmosphere (Figure 2a). The vigor of subsolidus mantle convection is controlled primarily by the viscosity of the mantle [109], and mantle viscosity is sensitive to temperature as well as the trace amount of water contained in nominally anhydrous minerals [110,111,112]; viscosity is lower at higher temperatures and at higher water contents. We thus expect rapid mantle convection when an early mantle was hot and wet. The presence of surface water makes plate tectonics possible [113,114], and rapid plate tectonics has long been considered to be essential for the removal of the massive amount of carbon dioxide in the atmosphere by the subduction of the carbonated seafloor [115,116]. Theoretical estimates suggest that such a sequestration of atmospheric carbon by rapid plate tectonics could have been completed in ∼100 million years [117]. The degassing of mantle water by plate tectonics takes more time [79], but by the end of the Hadean (4 Ga), the mantle probably dried out, and the Earth’s surface was covered by a deep ocean (∼6 km) [118].

During the Hadean, therefore, the surface environment changed drastically. During the first hundred million years or so, it was extremely hot (>200 °C) owing to the dense CO${}_{2}$-rich atmosphere, and more clement conditions were then reached by the sequestration of atmospheric carbon by plate tectonics [116]. In plate tectonics, water is degassed at mid-ocean ridges, but part of the surface water is returned to the mantle by the hydration of subducted materials [38,43,119]. When the tempo of plate tectonics is fast, degassing outweighs regassing, because plate motion is too fast for hydration to penetrate deeply [119]. The ocean deepens gradually by this imbalance of degassing and regassing. At the same time, the continental crust starts to grow by the melting of the subducted hydrated crust [120]. Competition between a deepening ocean and growing continents could result in the maximum exposure of continents in the mid-Hadean, potentially occupying ∼20% of the Earth’s surface, if we adopt the continental growth model of Guo and Korenaga [24] (Figure 2b). In addition, hotspot islands and oceanic plateaus are expected to have been more abundant than present because the hot early core results in a higher core heat flux and thus a greater flux of mantle plumes [121,122]. The Hadean Earth was also subjected to the bombardment of large left-over planetesimals [123,124], and an impact-generated topography (i.e., craters) can have an amplitude of a few kilometers [125]. Both mantle plumes and bolide impacts are at their most intense in the early Hadean and gradually decline afterwards. Due to fast plate tectonics, the topographies generated by these mechanisms would quickly (<10 million years) be subducted if formed on the seafloor.

The fast plate tectonics in the Hadean was made possible by the low viscosity of the hot and wet mantle [86]. As the mantle dries out, therefore, it convects more slowly. Plate tectonics in the Archean is characterized by slow plate motion [126,127], and the seafloor can persist for over 300 million years before being subducted. Slow plate tectonics allows for a more efficient hydration of the oceanic crust and lithospheric mantle, and regassing dominates over degassing, thereby starting the return of surface water into the mantle. The subduction of surface water takes place slowly, and the mean ocean depth decreases at a rate of ∼1 km per billion years [21]. With slow plate tectonics, the subsidence of the seafloor is affected considerably by the radiogenic heating in the mantle, which was greater in the past. At present, the seafloor subsides as it moves away from mid-ocean ridges, but in the early Archean, intense radiogenic heating in the mantle could halt or even reverse such subsidence [118]. As a result, the water-holding capacity of ocean basins is reduced, and the flooding of continents becomes more likely. At the same time, the seafloor becoming shallower allows hotspot islands, which usually become seamounts within ten million years or so, to be long-lived, in the order of a hundred million years. Thus, in the early Archean, such long-lived hotspot islands are probably the only exposed land [11]. Continents may have already been as massive as we see today [23,24], but all of them were below sea level (Figure 2c). The height of mountains resulting from continent–continent collision is limited in the early Earth because a hotter crust (owing to greater radiogenic heating) is much weaker, being unable to support large topographic variations [66]. As radiogenic heating declines, ocean basins can hold more water and mountains become higher, and combined with the subduction of water, continents become more likely to rise above sea level. The global and steady emergence of continents probably took place near the end of the Archean (2.5 Ga) [21,128,129], and the extent of exposed land has not changed much since then, except for occasional inundations caused by the fluctuations of plate motion [67,130] or by the influence of mantle density anomalies on surface topography [131,132,133].

## 3. Points of Discussion

### 3.1. Magma Ocean Solidification

The retention of most water in a solidifying magma ocean is a corollary of two factors: (1) a solid–melt mixture experiences a rheological transition at a solid fraction of ∼60% (e.g., [44,104]), and (2) a magma ocean after such a transition cools down due to the Rayleigh–Taylor instability (e.g., [45,97]). Neglecting these important processes would lead to the complete degassing of volatiles, as assumed in some models of magma ocean solidification [85,134].

The solidus and liquidus of the silicate mantle are separated by ∼1500 K in the lowermost mantle [105], but the melt fraction between them varies highly nonlinearly, and a solid fraction of 60% is reached only ∼200 K below the liquidus [97]. This effectively halts the cooling of the core in the very early stage of magma ocean solidification because, after the rheological transition, core cooling is modulated by the high solid viscosity of the overlying mantle layer. This situation contrasts with the earlier speculation that a magma ocean can quickly deplete the heat content of the core [91]. Inefficient core cooling in the beginning of Earth history allows high core heat flux in the subsequent stage of subsolidus mantle convection. High core heat flux helps to produce more numerous or stronger mantle plumes, thereby facilitating the formation of ocean islands.

### 3.2. Onset of Plate Tectonics

Some amount of water is of course degassed to the surface during magma ocean solidification. The presence of surface water is important as it allows the operation of plate tectonics (e.g., [114,119]). Without surface water, the mode of mantle convection is likely to be stagnant lid convection [135], which severely limits volatile exchange between the surface and the interior. In the geological literature, the notion of stagnant lid convection in the early Earth has been popular (e.g., [75,76,136,137,138]). However, its observational and theoretical basis is rather weak [79], and there are a growing number of observational studies that support the early onset of plate tectonics (e.g., [23,24,139,140,141,142]).

The early onset of plate tectonics is also necessary to sequester the massive amount of carbon dioxide in the early atmosphere [115,116,117], because the subduction of carbonated oceanic crust is probably the only means to do so. It has been suggested that, depending on the oxidation state of a magma ocean, a solidifying magma ocean could store a large amount of carbon as diamond [143], but this argument does not take into account that a solidifying magma ocean should experience frequent mixing due to the Rayleigh–Taylor instability.

### 3.3. Tempo of Plate Tectonics

As just noted, plate tectonics was required on the early Earth to sequester a large amount of atmospheric carbon. As long as surface water exists, the operation of plate tectonics is favored [144,145,146]. Additionally, the current understanding of rock mechanics indicates that the present-day operation of plate tectonics owes much to surface water [114], so given the likely presence of the Hadean ocean [147,148], it would be puzzling if plate tectonics was absent in the Hadean. Furthermore, recent models of continental growth suggest that the formation of continental crust was persistently active in the Hadean [23,24]. As plate tectonics is the only mechanism that allows the continuous production of continental crust [120] (it is possible to produce some amount of continental crust without subduction if it is only for a brief period [149,150]), the early onset of plate tectonics is consistent with these recent growth models as well.

As mentioned earlier, the viscosity of the mantle is a function of temperature and water content, and the vigor of mantle convection is determined by mantle viscosity. However, the viscosity of the mantle is spatially variable, and its relation to the vigor of mantle convection, or plate velocity, is not simple. Such a relation is influenced by the details of how degassing takes place during mantle melting [86,151], and the notion of fast plate tectonics in the Hadean and slow plate tectonics in the Archean is based on this sort of theoretical consideration [79]. Fast plate tectonics in the Hadean is also preferable from the perspective of atmospheric carbon sequestration [115,117]. Slow plate tectonics in the Archean has substantial observational support, including the thermal budget of Earth [152], the lifetime of passive margins [153], the cooling history of the upper mantle [53], the xenon isotopic composition of the present-day atmosphere [154], and the history of continental plate velocity [155,156].

### 3.4. Continental Growth

**Figure 3:**
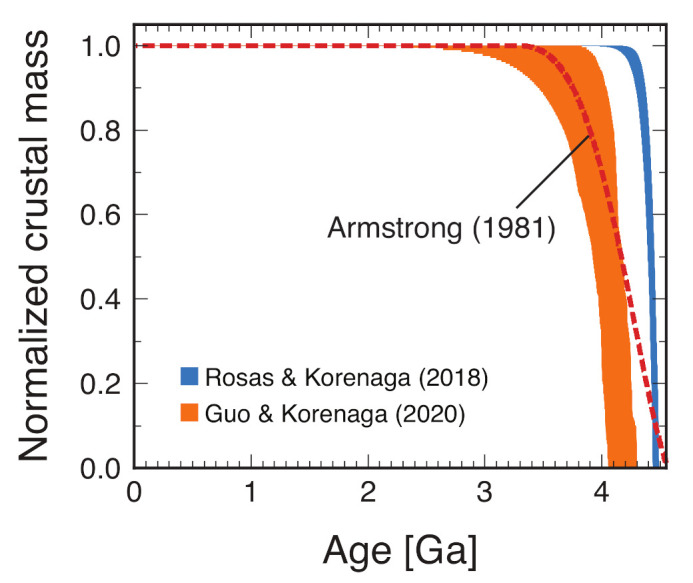
Continental growth models of Rosas and Korenaga [23] (blue) and Guo and Korenaga [24] (orange). Only middle 50 % of their solutions are shown for clarity. The model of Armstrong [54] (red dashed) is also shown for comparison. After [79].

The history of the continental crust on Earth—when it first appeared and how it grew—is as controversial as the onset of plate tectonics. The study of continental growth, however, has been unnecessarily confused in the last three decades or so, because it was not widely understood which aspect of continental growth was constrained by what kinds of observations [79,127]. In many growth models, virtually no continents existed in the Hadean, and the continental crust starts to grow substantially only from the mid-Archean (e.g., [55,58,59,157,158,159,160,161]). However, some of these models are limited to the extant continental crust (i.e., the crust that has been preserved to the present) and not about the history of the continental crust that existed in the past. Other models suffer from too narrow an exploration of the relevant model space. In the last decade or so, the growth model of Dhiume et al. [162], in which continents grow gradually to reach only one-quarter of the present-day level by the end of the Hadean and at three-quarters by the end of the Archean, has widely been popularized by these authors [76,163,164,165,166,167,168], but as pointed out recently [79,127,169], this model suffers from a fundamental logical flaw and has no observational basis. Armstrong [54,170] had long advocated that the presence of massive continents at the present-day level from the Hadean was consistent with available geological data (see also [171]), and recent studies corroborate this Armstrong model with new kinds of geochemical data from the coupled ${}^{146}$Sm-${}^{142}$Nd and ${}^{147}$Sm-${}^{143}$Nd system [23] and the ${}^{40}$K-${}^{40}$Ar system [24] (Figure 3).

Though the models of Rosas and Korenaga [23] and Guo and Korenaga [24] are both similar to the Armstrong model, their details in the Hadean differ considerably. In the model of Rosas and Korenaga [23], the continental crust starts to form right at ∼4.5 Ga, after magma ocean solidification, and it reaches the present-day level by ∼4.2 Ga. In the model of Guo and Korenaga [24], the start of crust formation is delayed to ∼4.3 Ga, and the present-day level is achieved at ∼4.0 Ga. As noted in a recent review [79], this discrepancy is not surprising because these models employ different assumptions on crust–mantle differentiation, and both models have some room for future improvement. If the model of Rosas and Korenaga [23] is closer to the truth, and if the early ocean was shallow, ∼40% of the Earth’s surface can be exposed land in the mid-Hadean. The value of ∼20% quoted in the previous section is meant to be a conservative estimate based on the model of Guo and Korenaga [24]. Additionally, these numbers (∼40% for Rosas and Korenaga [23] and ∼20% for Guo and Korenaga [24]) represent the maximum possible values in case of a relatively shallow ocean. If water degassing from rapid plate tectonics is efficient, the ocean could outgrow the continents, resulting in a mostly water world, with only ocean islands being exposed.

### 3.5. Mountain Ranges and Ocean Islands

The elevations of mountain ranges on continents of the early Earth were limited because the continental crust was relatively hot and thus weak. Heat arose from large amounts of radioactive isotopes in the continental crust [172,173]. High mountain ranges in the extant continental setting are supported by crustal thickening; for example, the crustal thickness of the Himalayan mountain range is about ∼70 km, whereas the average thickness of continental crust is ∼40 km [174]. When the crust was hotter and weaker, it became increasingly difficult to maintain such regional variations in crustal thickness [65,66].

Ocean islands made by hotspots are different from continental mountains. First of all, being the product of single-stage mantle melting, their crust is not as enriched in heat-producing elements as the continental crust [175,176]. Second, ocean islands are supported mostly by the flexure of the oceanic lithosphere (e.g., [177]). The strength of the oceanic lithosphere is primarily a function of the seafloor’s age [178,179], and ocean islands formed on reasonably old seafloors are firmly supported by the underlying lithosphere. Thus, hotspots erupted on the Archean seafloor, when slow plate tectonics is expected, can become high enough to become subaerial (like Hawaii and Tahiti), given a sufficiently strong mantle plume [63,180].

### 3.6. History of Ocean Volume

There was no direct method for observation of the volume of ocean in the past, and partly due to this, the ocean volume has often been assumed to be constant for the sake of simplicity (e.g., [33]). This is understandable because varying it without any observational constraints would be arbitrary. One indirect observation is that the record of frequent continental flooding preserved in the forms of sedimentary rocks on continents (e.g., Grand Canyon and Badlands). To explain such a sedimentary record, the mean height of continents must always be close to sea level so that continents can easily be flooded by fluctuations of the sea level [32]. The mean height of continents with respect to the mean sea level is called the continental freeboard, and the constancy of the continental freeboard (or equivalently, the constancy of sea level) is often interpreted to support the constancy of ocean volume (e.g., [38,181]). However, this use of freeboard neglects the time-varying nature of hypsometry. As mentioned in the introduction, the relative buoyancy of the continental domain with respect to the oceanic domain changes with time because all components in both domains change with the secular cooling of Earth. There have been many attempts to model the history of the continental freeboard (e.g., [33,34,35,36,37]), but it is only recently that all important variables have been included in modeling [21,118]. These recent models suggest that, to explain the constancy of the freeboard, long-term net water flux has to be at a rate of 3 to 4.5 $\times \;10^{14}$ g yr${}^{-1}$, which would translate to about twice as voluminous as the ocean in the early Archean, and that the global emergence of continents probably took place during the late Archean (∼2.5–3 Ga). Freeboard modeling depends critically on the assumption of a constant freeboard, which can be justified only back to ∼2.5 Ga [21,182], so the most conservative estimate is that the ocean was ∼50% more voluminous at 2.5 Ga. This level of water flux from the surface to the mantle has long been suggested by the degree of seafloor hydration (e.g., [38,43]). The theoretical estimate on continental emergence is also consistent with what recent geochemical studies suggest [128,129].

A recent study [183] suggested that the temperature dependency of water solubility in mantle minerals might be used to constrain the volume of the Archean ocean; the water capacity is lower for a hotter mantle, so some fraction of the water in the present-day mantle may not be retained in the past. This mineral physics constraint would be most effective if the present-day mantle is close to being saturated with water. Though there exist a range of estimates for the amount of water stored in the mantle (e.g., 0.2–1.6 ocean mass [184] and 10 ± 5 ocean mass [185]), the most likely value is about 1 ocean mass [21], which is well below the present-day saturation level (∼5 ocean mass) [183]. Nevertheless, the mineral physics constraint is useful in discounting the possibility of many oceans worth of water in the present-day mantle and thus an unrealistically deep (>10 km) ocean in the past.

### 3.7. Global vs. Local Emergence of Continents

As stated earlier, there is no definitive record for regionally expansive exposure horizons prior to 3.0 Ga, and this is based on the compilation of epicontinental sedimentary records for exposed areas larger than 30,000 km${}^{2}$ [21]; the oldest entry in this compilation is the ca. 2.9 Ga Mozaan Group in the Kaapvaal craton in South Africa [186,187,188]. The compilation includes only two other Neoarchean entries, the ca. 2.6 Ga Ghaap Group in the Kaapvaal craton [182,189,190,191] and the ca. 2.5 Ga Hamersley Group in the Pilbara Craton in Australia [192]. The ca. 3.0 Ga Pongola–Witwatersrand Basin in the Kaapvaal craton contains several weathering horizons [193,194,195], but only one of them is spatially expansive, as mentioned above. Numerous cherts of the 3.3–3.5 Ga Onverwacht Group cherts of the Barberton greenstone belt in South Africa have been suggested to result from continental weathering [196], but this by itself does not constrain the spatial extent of exposed continental crust. For comparison, the ca. 3.48 Ga Dresser Formation in the Pilbara craton is approximately only 30 km long.

A substantial amount of exposed land may be inferred even from highly localized geological data, if such data can constrain the chemical or isotopic composition of sea water, which could reflect the extent of continental weathering [128,129,197]. This geochemical approach is promising, though the interpretation of relevant data always involves geochemical reasoning or modeling, which may not be sufficiently robust. For example, the work of Johnson and Wing [129] is based on the oxygen isotopic composition of sea water, the interpretation of which is still controversial [198,199].

### 3.8. Comments on Russell (2021)

Recently, Russell [14] criticized the importance of exposed land during the origins of life, stating that it is geologically unfounded. Although his motivation to defend the hypothesis that life began at submarine alkaline vents is understandable, his arguments against exposed land on the early Earth warrant some examination. He stated “the evidence refuting the warm little pond scheme is overwhelming given the facts that (i) the early Earth was a water world, (ii) its all-enveloping ocean was never less than 4 km deep, (iii) there were no figurative ‘Icelands’ or ‘Hawaiis’, nor even an ‘Ontong Java’ then because (iv) the solidifying magma ocean beneath was still too mushy to support such salient loadings on the oceanic crust”. These four points are discussed in the following.

The first and second points are actually the same, and to support the notion of a 4 km-deep ocean, Russell cited the following ten references: Morbidelli et al. [200], Bounama et al. [201], Valley et al. [202], Cavosie et al. [28], Pope et al. [203], O’Neil et al. [27], Korenaga et al. [21], Genda [204], Ueda and Shibuya [205], and Johnson and Wing [129]. However, none of these references can be used for his purpose. Morbidelli et al. [200] describe the delivery of water to Earth during planetary formation and do not provide a good constraint on the amount of water, let alone the distribution of delivered water in the different parts of Earth, e.g., the surface, the mantle, and the core. The work of Bounama et al. [201] is just a modeling study on the history of mantle degassing and regassing using assumed parameterizations. This type of modeling, which can be traced back to the classic work of McGovern and Schubert [39], depends critically on assumptions. Many of the assumptions made by Bounama et al. [201], such as the scaling of mantle convection, are questionable (see a review of this type of modeling studies in Section 3 of Korenaga’s work [206]). Valley et al. [202] and Cavosie et al. [28] both provide reviews of Hadean zircon geochemistry in support of the presence of an ocean in the Hadean but do not mention the depth of the ocean; zircon geochemistry is insensitive to such a physical aspect. In fact, the oxygen isotopes of Hadean zircons themselves do not demand the presence of a globally connected ocean ([29], Section 9.12), though the presence of hydrated mineral inclusions in those zircons is consistent with a spatially expansive ocean [148]. Pope et al. [203] suggested that, based on the hydrogen isotope of 3.8 Ga serpentines, the ocean was up to 26 % more voluminous back then. This volume of the early Archean ocean is actually smaller than suggested from freeboard modeling, but as noted by Korenaga et al. [21] (see Section 4d), this hydrogen isotope study is problematic in at least a few aspects. The work of O’Neil et al. [27] is about the felsic Archean crust forming from mafic Hadean crust. The formation of the felsic crust likely involves water [120]; thus, the presence of an ocean is implied, but no constraint on ocean depth can be gained from such a petrological study. The work of Korenaga et al. [21] contains a review of the present-day water budget and deep water cycle, freeboard modeling, and the compilation of geological records relevant to the extent of exposed land mass, but as mentioned earlier, it does not provide firm constraints on the early Archean and Hadean. Genda [204] provides a review of the origin of Earth’s water, with a spirit similar to that of Morbidelli et al. [200], because they are both experts of planetary formation processes. Ueda and Shibuya [205] present an experimental study of water–rock reactions that may have taken place in the early ocean, and as such, they do not provide any constraint on ocean volume. Johnson and Wing [129] report an estimated seawater oxygen isotope composition at 3.24 Ga, and based on the modeling of their data and other published isotope data, suggest the emergence of continents sometime between 3 and 2.5 Ga. As mentioned in Section 3.6, this study is consistent with the freeboard modeling of Korenaga et al. [21] and Rosas and Korenaga [118], but this does not negate the possibility of ocean islands in the Archean, nor the possibility of a shallow ocean in the Hadean.

The third and fourth points of Russell [14] are also basically the same, and for the notion of the mushy Hadean surface, he cites the work of Monteux et al. [207], which advocates a prolonged (>500 million years) duration of magma ocean solidification. This work is problematic in at least two major aspects. First, their melting model is based on the experimental study of Andrault et al. [208], which has recently been suggested to be grossly in conflict with other experimental data [209]. Second, their cooling model does not take into account the role of the Rayleigh–Taylor instability, which can efficiently cool down a solidifying mantle ocean [97,104].

Russell [14] touched on continents only in passing as “While it is admitted by Damer and Deamer [8] [in their presentation of an alternative hypothesis of an origin of life in hydrothermal fields on land] that the Hadean Earth did not have continents,…”. Actually, Damer and Deamer [8] did not state that continents would be absent in the Hadean. They only suggested that, based on the work of Van Kranendonk [210] and Bada and Korenaga [11], volcanoes emerging through a global ocean would be the original landmass on the Hadean Earth. As discussed in Section 3.4, the growth of continents is yet to be fully understood by geologists, but the possibility of massive continents in the Hadean is difficult to discount.

## 4. Summary

In this review, I have tried to provide a concise overview for a likely landscape in the early Earth, by assembling recent developments in earth sciences with the help of a geodynamically reasonable framework. We can expect that ocean islands have always provided a limited amount of exposed land through Earth history. In addition, there is a possibility of massive exposed continents in the mid-Hadean if continental growth was rapid and ocean deepening was slow. As can be understood from the previous section, many aspects of the suggested scenario suffer from large uncertainties, but interdependence among the different components of the Earth system and its temporal continuity would help us evaluate a certain possibility in the proper context. For example, the depth of the very early ocean is tightly connected to how a magma ocean solidifies, which also affects the tempo of Hadean plate tectonics. Additionally, it is critical to know that studies on continental growth have witnessed a drastic turnover in the last few years and that our understanding of magma ocean dynamics is still in a state of flux.

What is most certain is, however, the nearly constant presence of oceanic islands throughout Earth history, including the Hadean [11]. Our understanding of the cooling history of Earth’s core indicates steady core heat flux, which manifests itself as rising plumes in the mantle and hotspot islands on the surface. Therefore, abiogenesis theories based on wet–dry cycles on exposed land, such as the hot spring hypothesis of Damer and Deamer [8], are not without geological support. However, the spatial extent of such oceanic islands is likely to have been as limited as in the present, and it is unclear that such a limited amount of land is sufficient to foster prebiotic evolution. At the moment, abiogenesis theories that allow such quantitative discussions appear to be lacking. In summary, the existence and role of exposed land during the period suggested for the emergence of life remains an open question.
